# Recommendations for the creation of benchmark datasets for reproducible artificial intelligence in radiology

**DOI:** 10.1186/s13244-024-01833-2

**Published:** 2024-10-14

**Authors:** Nikos Sourlos, Rozemarijn Vliegenthart, Joao Santinha, Michail E. Klontzas, Renato Cuocolo, Merel Huisman, Peter van Ooijen

**Affiliations:** 1 Department of Radiology, University Medical Center of Groningen, Groningen, The Netherlands; 2 DataScience Center in Health, University Medical Center Groningen, Groningen, The Netherlands; 3 Digital Surgery LAB, Champalimaud Foundation, Champalimaud Clinical Centre, Lisbon, Portugal; 4 Department of Medical Imaging, University Hospital of Heraklion, Heraklion, Greece; 5 Department of Radiology, School of Medicine, University of Crete, Heraklion, Greece; 6 Department of Medicine, Surgery, and Dentistry, University of Salerno, Baronissi, Italy; 7 Department of Radiology and Nuclear Medicine, Radboud University Medical Center, Nijmegen, The Netherlands; 8 Department of Radiation Oncology, University Medical Center Groningen, Groningen, The Netherlands

**Keywords:** Benchmark dataset, Validation, Bias, Artificial intelligence (AI) software, Radiology

## Abstract

**Abstract:**

Various healthcare domains have witnessed successful preliminary implementation of artificial intelligence (AI) solutions, including radiology, though limited generalizability hinders their widespread adoption. Currently, most research groups and industry have limited access to the data needed for external validation studies. The creation and accessibility of benchmark datasets to validate such solutions represents a critical step towards generalizability, for which an array of aspects ranging from preprocessing to regulatory issues and biostatistical principles come into play. In this article, the authors provide recommendations for the creation of benchmark datasets in radiology, explain current limitations in this realm, and explore potential new approaches.

**Clinical relevance statement:**

Benchmark datasets, facilitating validation of AI software performance can contribute to the adoption of AI in clinical practice.

**Key Points:**

- Benchmark datasets are essential for the validation of AI software performance.
- Factors like image quality and representativeness of cases should be considered.
- Benchmark datasets can help adoption by increasing the trustworthiness and robustness of AI.

**Graphical Abstract:**

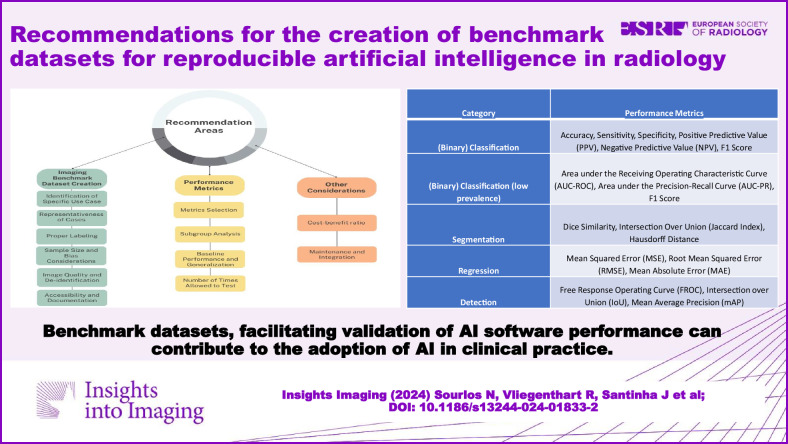

## Introduction

The development of artificial intelligence (AI) algorithms in healthcare has gained significant momentum in recent years, including in radiology. In early 2023, there were more than 200 commercially available AI software solutions for radiology alone [1]. One important aspect of external validation of AI models is the creation of benchmark datasets. A benchmark dataset is a well-curated collection of expert-labeled data that represents the entire spectrum of diseases of interest and reflects the diversity of the targeted population and variation in data collection systems and methods. Such datasets are vital for validating, in the sense of establishing the reliability and accuracy of, AI models, increasing trustworthiness, and the chance of robust performance in real-world applications [2–4].

If the dataset used to develop and validate an AI algorithm is not representative of the target population, biases could arise that could have severe consequences for a large group of patients [3]. For instance, if a dataset is derived from a relatively homogenous source population from within a well-established healthcare system, the developed algorithms may not generalize effectively to, for example, a limited-resource setting with different demographic and pathophysiological features of the population. This may further amplify health inequities, potentially leading to worse healthcare outcomes for those marginalized populations [5]. Also, algorithms developed on over-used public datasets derived from a hospital population may exhibit subpar performance if applied in a screening setting on individuals with similar demographics but different disease prevalence [6, 7]. This could lead to missed diagnoses on a large scale, especially in the light of automation bias [8]. Logullo et al [9] reviewed studies in which AI was trained to diagnose lung nodules (detect, segment, or classify them) using public datasets. They showed that 49% of their included studies used LIDC-IDRI [10] or LUNA [11] or a dataset derived from them during model development and/or validation. The characteristics of such public datasets might differ from those of the intended use case of an AI algorithm that utilized them for training/validation. For example, the volume quantification of nodules might have been derived from manual diameter measurements, which will give different results compared to fully automated measurements. In addition, these public datasets might have been preprocessed and their quality might differ from those used in clinical practice. It is therefore essential to perform further analysis to ensure the clinical utility of the dataset prior to deciding if it should be used for the particular task of interest.

It is imperative to create and enable access to benchmark datasets encompassing diverse populations and disease characteristics to validate the performance of an AI algorithm and test its generalizability. Moreover, the benchmark creation process must be transparent and rigorously documented. Furthermore, the dataset should be representative of the clinical context it is designed to address (e.g., screening and clinical diagnosis). Consequently, creating a benchmark dataset is not a straightforward task, as biases could arise in various steps in its formation process [3]. Factors to limit bias include the data sources used, anonymization steps, data format, and annotation methods.

There are initiatives to standardize infrastructure for validating AI software in imaging, enhancing transparency [1]. Furthermore, recommendations for a benchmark dataset for medical imaging in screening settings exist, but no standardized approach for clinical applications [12]. In pathology, proposals for creating test datasets to validate AI performance are already in place [13]. For more general AI solutions, it might be argued that local fine-tuning of a model and strict post-market surveillance is most efficient since data are scarce. However, before model deployment, the models’ weaknesses need to be established before introduction in the clinic, especially in rare diseases.

**Fig. 1:**
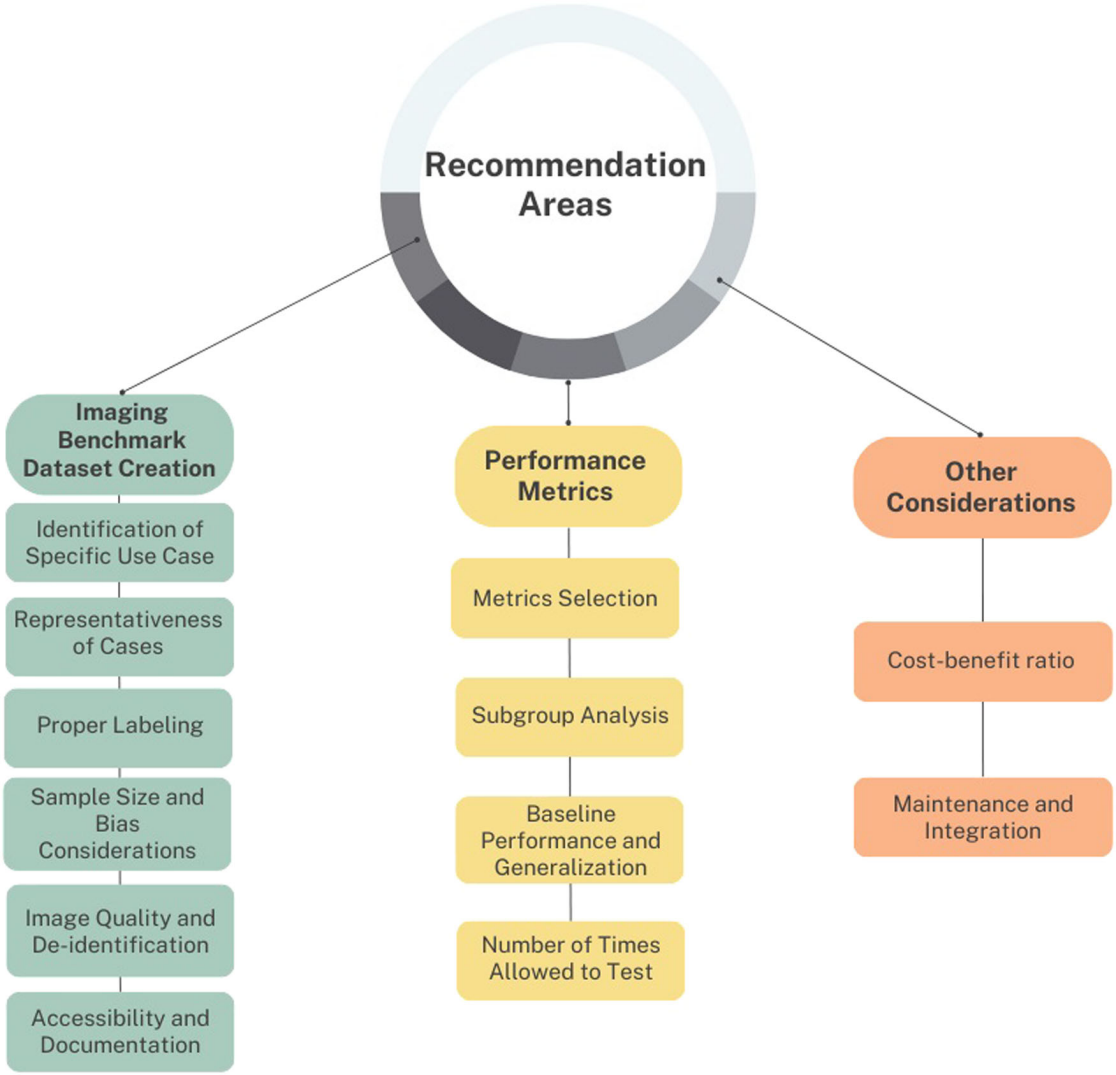
Considerations for the creation of a benchmark dataset

This paper explores the key considerations in creating imaging benchmark datasets (Fig. 1) to validate the performance of AI software, addressing challenges like data quality and data heterogeneity, and emphasizing domain experts’ input. Finally, it discusses metrics for evaluating model performance and provides recommendations for creating benchmark datasets in clinical practice. The primary objective of this paper is to guide the development of these datasets for AI software assessment in hospitals.

### Imaging benchmark dataset creation

When developing a benchmark dataset, there are several steps to be taken [4, 12]. The following section highlights and examines the most crucial of these steps.

#### Identification of specific use case

It is essential to identify the specific use case(s) prior to creating a benchmark dataset. This involves considering various tasks such as object detection, binary or multiclass classification, segmentation, and regression, and their requirements (e.g., correct bounding box for detection, correct contour for segmentations, etc.). The clinical context, including the disease(s) of interest, modality, target population, and healthcare setting, should be clear, such as detecting chest X-ray abnormalities vs a normal chest X-ray in patients presenting to the emergency department of a secondary or tertiary referral center. Furthermore, it is important to identify the most accurate ground truth labels. In many cases the expert user is regarded as the ground truth, but more on practical grounds than based on actual proof. Follow-up of patients or more extensive diagnostics are often lacking resulting in the absence of a definitive ground truth. For example, biopsy results should be preferred to clinical observations to decide if a lung nodule is malignant, but they are either not available at all (yet) or just not included in the data collection. Furthermore, in this case, the required 2-year follow-up data that could be used to confirm the benign nature of nodules is also often lacking.

#### Representativeness of cases

A crucial aspect to consider is the representativeness of cases encountered in clinical practice. The dataset must reflect real-world scenarios, including the disease severity spectrum, and ensure diversity in terms of demographics, vendors, and other factors.

One challenge that is difficult to address is the inclusion of rare diseases. Given their low prevalence, a very large sample size would be needed for these cases to be properly represented. Since it is commonly unfeasible to acquire a sufficiently large dataset, one proposed method is augmenting the dataset by generating synthetic data including variants of the underrepresented subsets [14]. For segmentation tasks, the inclusion of synthetic cases has been shown to lead to an improvement of the intersection over union (IoU) of up to 30% [15]. For detection tasks like that of the chromophobe subtype, synthetic histology images improved accuracy in clinical settings [16]. However, potential biases introduced by synthetic dataset heterogeneity in clinical practice are still under research [17].

Considering all the above-mentioned factors (spectrum of disease, diverse demographics, etc.) will help guarantee that the dataset is representative of the patient population and the intended clinical setting (e.g., primary care, public hospital, academic centers, or population screening).

For instance, a dataset derived from a population-based screening cohort is unsuitable for validating algorithms intended for routine computed tomography (CT) scans of the hospital population due to differences in scan protocols and disease prevalence. Validating algorithms is challenging due to clinical indication heterogeneity and incidental findings leading to new diagnoses, especially in broader clinical settings like abdominal CT scans. In these cases, there may be patients with varying indications ranging from analysis of an incidental finding to periodic oncologic follow-up [18, 19]. This is why it may be more straightforward to implement or evaluate AI techniques in highly specialized environments characterized by well-defined indications and a limited spectrum of findings, such as mammography screening [20, 21] and prostate cancer detection on MRI [22].

An example of a non-representative dataset in terms of population characteristics is the MIMIC-CXR dataset [23, 24], which consists mainly of data from a single hospital’s emergency department [6]. MIMIC-CXR is a large-scale dataset of chest X-ray images with associated radiology reports. For chest CT for lung nodule detection tasks well-known datasets are the LIDC-IDRI [10] and its derived LUNA16 [11]. Their popularity among researchers is due to being the only publicly available datasets providing lung nodule coordinates. However, AI solutions based on these datasets may have limited generalizability. A study by Li et al [25] showed that algorithms trained on independent datasets and LUNA16 maintained high performance when tested on a non-LIDC-IDRI dataset. In contrast to that study, Ahluwalia et al [6] showed that when chest radiograph classifiers are validated in a geographically and temporally different real-world dataset their diagnostic performance may drop in certain subgroups. Thus, caution must be exercised when applying a solution developed based only on, for example, the public LUNA16 dataset to real-world scenarios.

#### Proper labeling

The main characteristic of a well-curated benchmark dataset is that it should be properly labeled to be used as a reference standard for validation studies, ideally by having sufficiently long follow-up, or pathological proof (biopsy and/or histology). Often, reader consensus or majority voting is taken as a proxy, since histology or cross-sectional imaging of all participants is usually not available in a retrospective study design, nor ethically feasible in a prospective setting. This (inherently imperfect) approach requires the involvement of domain experts, including radiologists. The years of experience of these experts should be considered and reported, and cases with poor interobserver agreement should be identified and analyzed for any (systematic) errors. Another consideration related to the labeling process is the types of labels that should be accompanied by proper instructions, especially when these labels are collected from different hospitals, to ensure homogeneous results. It is also crucial to decide on the annotation format like DICOM (DICOM-SEG, RTSTRUCT), NIfTI, or BIDS [26]. For ultrasound images, any image annotation format that either preserves the grayscale image or the RGB colors is sufficient [27]. Diaz et al [26] provided a comprehensive guide of (open access) data curation tools and Willemink et al [28] presented a list of steps for preprocessing medical imaging data and explained the difficulties in data curation and data availability.

Another important consideration is the types of metadata that should be included along with the annotations. Metadata can include information such as de-identified patient demographics, relevant clinical history, etc., which can help contextualize the labeled data and provide useful information for downstream analysis. The inclusion and analysis of metadata should be done with caution since there might be correlations between metadata of different formats [29]. In addition to the above, metadata should also reflect the information available to an AI model in clinical practice, if it is to be used directly for inference in clinical cases [30]. At last, it is possible to include in metadata (like in DICOM-SEG), information on whether the labels were obtained manually, semi-automated, or fully automated using an AI algorithm, to ensure anonymity, as well as to allow the evaluation of inter and intra-observer variability. For cases with multiple binary segmentations (e.g., one from each radiologist) some approaches that can be used to select the input mask to an AI algorithm are taking the intersection of the masks, their mean, their union, or randomly selecting one of them. It is also possible to perform a majority vote on a pixel basis [31]. The above methods are two-stage approaches in which curated labels are created based on the available ones [32]. There is also the need to provide specific recommendations on how to deal with regions where radiologists are uncertain if they belong to a tumor or not [33].

Of equal importance to the type and format of metadata is the issue of data harmonization. Data collected from multiple centers is needed to enhance stability and robustness but exhibit variations in clinical and/or imaging characteristics obtained from diverse scanners and protocols [34]. Common harmonization techniques for tabular data include standard scaling and ComBat [35], whereas histogram equalization, adaptive histogram equalization, and contrast-limited adaptive histogram equalization are commonly used to harmonize medical images [36]. There are still open research questions regarding the limitations of reproducibility of harmonization methods, especially when the variations are related to radiomic features [37]. For example, the ComBat harmonization is a statistical method developed to remove the batch effects in microarray expression. However, unlike gene expression arrays for which ComBat was designed, radiomic features have different complexity levels, which are expected to be non-uniformly affected by variations in imaging parameters [38]. Furthermore, ComBat harmonization aims only to remove the variance attributed to the batch effects while maintaining the biological information, but using ComBat to correct these effects directly on patient data without providing the correct biological covariates that actually do have an effect on radiomic feature values will lead to a loss of biological signals. This is because ComBat will assume that the variations in radiomic feature values are only attributed to the defined batch, and thus would not perform uniformly [39]. For the above reasons, ComBat corrections cannot just be applied during inference, and it rather requires both the training and test data to be processed together by a model, changing the feature values as well. Even in the case of a single participant, the entire harmonization process should be repeated from scratch, and the model would have to be retrained as well. Therefore, the ComBat method cannot deal with prospective data (impractical to be used in clinical settings), since its performance depends on variations between batches, making its use not optimal and not applicable to clinical practice [39–42]. Currently, European Horizon 2020 projects work on data harmonization methods [43]. One of them is the ChAImeleon project [44] which recently announced a challenge in which harmonized multimodality imaging and clinical data will be provided for many types of cancer, allowing development and comparison of algorithms.

#### Sample size and bias considerations

A benchmark dataset should be appropriately sized for the task at hand, and should consider the clinically relevant difference in effect size, and the desired level of statistical significance to be achieved. Preferably, sample size calculations are performed, although no standardized method is available for modern AI tools to date due to their complexity [45, 46]. Generic sample size calculations can be performed in cases where areas under the curve (AUCs) are calculated, with a minimum sample size for a given AUC, confidence interval, and confidence level provided [47]. A review performed by Balki et al [46] showed that evidence of sample size calculations is scarce in AI applications in medical research. Only a few studies performed any kind of sample size analysis. Rajput et al [48] showed that to consider the sample size adequate, the classification accuracy of a model should be above 80% and the effect size should be bigger than 0.5 according to Cohen’s scale. For sample size calculation of the validation dataset, Goldenholz et al [45] developed a model-agnostic open-source tool that can be used for any clinical validation study. Balki et al showed that both pre- and post-hoc methods have their strengths and weaknesses and advise that researchers should try to perform both to estimate sample sizes or consult a biostatistical when conceptualizing a study [46]. It should also be noted that the choice of sample size also depends on the algorithm that will be used. More complex models (based on deep learning (DL)) usually require more data compared to machine learning algorithms (e.g., decision trees). As traditional sample size estimations cannot derive a conclusion regarding the clinical value of a machine learning algorithm due to its complexity; tools like sample size analysis for machine learning can be useful [45]. Using this tool, by specifying the performance metrics to calculate, and some other parameters such as the required precision, accuracy, and the ‘coverage probability’, an estimation of the minimum sample size required to achieve metric values above a cut-off value can be provided. For machine learning solutions other than predictive models there is still no consensus on the sample size, but the more variables and the rarer the outcome, the larger the sample size needed.

The availability of resources to collect the data, and the rarity of diseases of interest, may limit the number of cases unless the dataset is augmented. The dataset’s balance*—*whether maintaining natural disease prevalence or having equal normal and disease cases*—*depends on its intended use. If the dataset will be used to validate the real-world applicability of an AI algorithm, then the natural disease prevalence as present in the target population should be maintained. If the dataset’s purpose is to be used to train machine learning algorithms, then a balanced dataset is preferable since otherwise a very large sample size is needed to obtain optimal performance. Furthermore, it is not guaranteed that increasing the sample size will lead to a more accurate AI algorithm, as demonstrated in the case of distinguishing various clinical conditions that could indicate the presence of prodromal Alzheimer’s disease [49]. Efforts should be made to ensure that the risk of bias is low by considering possible factors of bias during dataset creation [30].

One dataset that is frequently used in the literature [50, 51] as an external validation for AI tools is the MIMIC-CXR dataset [22, 23]. Caution should be given to the fact that it consists of single-center data and might not be representative of geographically different populations. A study by Ahluwalia et al [6] showed that if a subgroup analysis is performed, the performance of chest radiograph classifiers is dependent on patient characteristics, clinical setting, and pathology. Still, the creation of such large databases can facilitate progress in creating AI solutions that could potentially be implemented in clinical practice and should be promoted, especially given the fact that they are still largely lacking for other imaging modalities like CT, MRI, and PET/CT. Many other forms of bias can arise during the data collection and annotation phases. A detailed overview is provided in a recent review [3].

#### Image quality and de-identification

When creating a benchmark dataset in radiology, image quality is crucial. Images must be free of artifacts that render them undiagnostic and should be correctly preprocessed [28]. Furthermore, to ensure reproducibility, any preprocessing of the images (e.g., noise reduction, intensity normalization, or augmentation) should be thoroughly described and the software (code) used should be made available to the researchers who will perform the validation. Images in a benchmark dataset should be acquired using appropriate acquisition settings and parameters, similar to those of the intended use. Be aware that images from older scanners in open datasets might differ from current clinical practice, making them unsuitable for benchmarks. Detecting the performance drift of an algorithm that was trained with such images, can be done with different methods such as just using the scan date to exclude them or unsupervised prediction alignment [52] to correct for that drift. Other methods include checking the metadata for parameters that indicate the year of the scanner, or the image quality of the scans and confirming that it is not of low resolution, that there are no signs of degradation, and that there are normal levels of noise present. Apart from the above, data drift can also be caused by changes in clinical population (demographic or disease prevalence changes), and/or changes in clinical guidelines, diagnostic criteria, and treatment protocols used in clinical practice. Therefore, these factors should always be assessed to evaluate if a data drift occurred.’

Data privacy and security are legally required in healthcare. Protection of personal data can be achieved through different techniques like randomization (deletion of identifiers), cryptographic techniques, restricted access, etc. [53], which also must comply with relevant regulations. In the case of a restricted dataset, hosting it using privacy-preserving techniques (e.g., encryption) can ensure the protection of sensitive information.

In the European Union (EU), privacy and security laws, especially Europe’s General Data Protection Regulation (GDPR), do not allow unrestricted data sharing with other institutions to improve models. Even with de-identified metadata, it has been shown that it is for example still possible to reconstruct the face of the individual who underwent an MRI scan of the head [54]. One promising solution to the privacy preservation issue is federated learning (FL) strategies, where the model is brought to data from different institutions (and therefore heterogeneous patient data) to train and test without compromising privacy and security as the data do not leave the center’s server [55, 56]. In the case of FL, as image and label quality verifications cannot be done in a centralized approach, data quality becomes the sole responsibility of the data-providing institution. At last, special caution should be taken for cases in which patient data are burnt in the DICOM images and/or secondary captures. Some methods to automate the process of removing burnt patient data exist [57], but manual intervention might still be needed to confirm the correctness of these methods.

#### Accessibility and documentation

An important concern when developing benchmark datasets, taking the findable, accessible, interoperable, and reusable principles into account [58], is easy accessibility for researchers. The dataset should come with a metadata file containing the information needed to access and handle data. Moreover, the manuscript describing the dataset and possible use cases should follow specific reporting guidelines appropriate to the type of application [59]. Relevant clinical and demographic information should also be made available to allow subgroup analysis [60].

Xie et al [61] used the MIMIC-IV-ED database [62] to create a publicly available benchmark dataset of electronic health records of more than 400,000 adults admitted to the emergency department of a hospital. By making such a large dataset publicly available, they stimulate other researchers and companies to use that database to develop and test their solutions. Another example of a large dataset available to researchers is the NLST dataset [63]. The dataset consists of either low-dose CT scans or chest radiographs, along with accompanying clinical data. It is maintained and can be accessed through the cancer imaging archive [64]. Both these datasets are easily accessible and are accompanied by participants’ clinical and demographic characteristics.

### Performance metrics

Apart from dataset creation considerations, different aspects of the performance metrics chosen to evaluate the model should also be taken into account. Performance metrics help identify the inherent weaknesses of the model that could cause bias.

#### Performance metrics selection

The selection of performance metrics is crucial in assessing an algorithm’s performance on a benchmark dataset in radiology. Performance metric selection depends on the model’s objectives or desired outcomes, and different metrics may be more appropriate for different tasks [65, 66]. For example, metrics such as sensitivity and negative predictive value (NPV) are relevant for a dataset designed for screening purposes given the low prevalence of disease. Providing recommendations on metrics’ relevance in clinical scenarios can improve dataset usage and awareness of pitfalls [65].

For most clinical tasks, multiple performance metrics should be reported to give an overall impression of the model performance, including its inherent errors given a specific clinical setting (e.g., low prevalence) [67]. It should be ensured that they provide clinically relevant information that is easily interpretable by the end-user. Table 1 shows some commonly used performance metrics and their categories.

**Table 1:** Commonly used performance metrics and their categories [67, 96]

Category	Performance metrics
(Binary) Classification	Accuracy, sensitivity, specificity, positive predictive value, NPV, and F1 score
(Binary) Classification (low prevalence)	The area under the receiving operating characteristic curve (AUC-ROC), the area under the precision-recall curve, and the F1 score
Segmentation	Dice similarity, IoU (Jaccard Index), and Hausdorff distance
Regression	Mean squared error, root mean squared error, and mean absolute error
Detection	Free response operating curve, IoU, and mean average precision (mAP)

Importantly, some of those metrics like the AUC-ROC and accuracy derived from a balanced dataset do not directly translate to low prevalence settings due to the naturally large proportion of false negatives, even in a poor classifier. It should be noted that the same metric may be referred to in different ways based on the domain it is applied to. For example, the Dice coefficient could be the same as the F1 score for segmentation, or recall could be the same as sensitivity depending on the profession of the end-user. It is also recommended to report confidence intervals since they are of high importance for performance metrics in biomedical research due to the extra information they provide for the samples used [68].

#### Subgroup analysis

Reporting performance metrics for subgroups, such as by age, sex, or race [5, 69, 70], can help to assess bias and identify specific subgroups in which the model might underperform. It should be noted that GDPR does not allow requests from participants to declare their race unless this is the study’s primary goal, limiting the possibilities for this subgroup analysis. A workaround can be using summary demographics at a group level, which has disadvantages. Tripathi et al [71] reviewed publicly accessible imaging datasets and found that there are many issues related to, among others, demographics, race, and geographic diversity of different populations.

Tools like Aequitas [72], and FUTURE-AI [73], can help to analyze the fairness and bias of models and provide guidance on how to address any issues that arise, and PROBAST-AI [30, 74] will provide guidelines on assessing the risk of bias. However, the final version of PROBAST-AI has not yet been published [59]. Regulations and recommendations on how to avoid biases can be found in the European Parliament’s document for AI in healthcare [75]. Furthermore, information about specific subgroups and the data used to develop an algorithm can be provided through Model Cards [76], helping to enhance transparency and accountability in model deployment.

It is beneficial to utilize a benchmark dataset to evaluate the presence of bias within specific subgroups of the populations mentioned above. However, in addition to this approach, various techniques can be employed during the development and post-processing of the model to mitigate these biases [77] such as generative AI techniques to augment the training data. For instance, Burlina et al [78] demonstrated that by generating synthetic fundus images of the eye, the discrepancies between individuals with dark and light skin tones were minimized. Another approach is the application of adversarial methods, which not only enhance a model’s performance on a specific variable of interest but also minimize the ability of a second model to correctly identify protected attributes from the features learned by the first model [79]. Li et al [80] successfully demonstrated this approach for skin lesion classification. Finally, model predictions can be calibrated across different subgroups as part of the post-processing stage. Ultimately, the effectiveness of these methods can be assessed by comparing the model’s performance to the benchmark dataset, which can also include examples generated with these techniques (e.g., synthetic images).

#### Baseline performance and generalization

Establishing and reporting a reference (baseline) performance based on criteria set by clinicians on how well a model should perform on a particular task of interest, can provide context for the lower bound of required performance. Comparing AI software’s performance on benchmark datasets with that of radiologists or other expert clinicians reveals areas where AI or clinicians are superior, indicating the potential added value of the software. For example, for medical images, a carefully designed study, e.g., according to the multi-reader multi-case design, is recommended to establish if AI could be beneficial, although this could be very resource-intensive given the number of human readers required [81].

The comparison of the AI performance vs clinical experts is challenging due to the fact that the clinically preferred settings of the algorithm depend on the context. Efforts to create open-source datasets include the WILDS benchmark dataset [82], aiming to address naturally occurring distribution shifts (changes in imaging characteristics) in a diverse set of problems (e.g., in tumor identification tasks across different acquisition sites), BenchMD for variations across hospitals [83], and the DomainBed suite [84], consisting of multi-domain datasets, and focusing on assessing the generalizability of AI algorithms in real-world settings. Another great resource of publicly available datasets, along with their performance on a dataset of interest can be found in the papers with code website [85], and datasets focusing on medical imaging tasks in the GitHub repository of Adalca [86]. After establishing a baseline performance on an open-source dataset, a restricted-access benchmark dataset that has not been used for model development can then be utilized to get an estimate of the true performance of the developed AI algorithm in new, unseen cases.

Another way to assess the limitations of an algorithm developed on a different source population is to conduct a failure analysis [4] using a benchmark dataset. Oakden-Rayner et al [87] evaluated the performance of a DL model designed to detect fractures on X-rays. Even though the model maintained a very good performance during external validation, an algorithmic audit revealed an elevated error rate in unexpected edge cases, such as Paget’s disease, along with a significant alteration in the model’s operating point.

#### Number of times allowed to test

Finally, it is essential to consider how many times an algorithm is allowed to run on the same benchmark dataset used for external validation only [12]. Providing the dataset and allowing many evaluations of the algorithm in the benchmark dataset can increase the risk of overfitting, resulting in misleading performance results. Establishing a limit to the number of runs or providing a different fraction of the dataset in each test run can help mitigate that risk. Ideally, the benchmark dataset should not be directly accessible to the users and the specific cases used during validation should be selected randomly each time (given that the dataset size allows that). A study by Roelofs et al [88] demonstrated, that contrary to popular belief, when a separate test set is used only once to obtain the final ranking in Kaggle competitions (although a holdout set with similar characteristics could have been used multiple times for the public ranking), there were limited indications of significant overfitting, showing that the test set could potentially be used multiple times. At last, an agreement should be achieved prior to performing the validation on where the results would be available (peer-reviewed journal, website, etc.) and ensure that they are reported correctly using the designated reporting guidelines [59].

### Other considerations before creating and using a benchmark dataset

Apart from the dataset and the performance metrics used to evaluate a software’s performance, other factors can affect the creation and use of a benchmark dataset. These are listed below.

#### Regulatory compliance

Creating a benchmark dataset requires adherence to regulations like GDPR or HIPAA [89], ensuring data privacy and security, and addressing ethical considerations such as transparency and fairness. These regulations evolve constantly (upcoming AI Act in EU [73]) necessitating regular dataset updates and maintenance. This involves allocating resources and expertise in regulatory compliance throughout the workflow, from data acquisition to reporting validation results. It is also important to thoroughly vet the privileges and access granted to the software provider when validating their software to ensure no compromise of patient privacy and security. This can also be achieved by installing the software locally and granting it access to the data offline or by using encryption. Moreover, compliance with local Institutional Review Board regulations must be achieved prior to using patient data for model development/validation.

#### Maintenance and integration

Providing the dataset together with technical support is essential. This includes assistance with software installation and evaluating algorithms against the benchmark. Furthermore, the dataset’s interoperability with various picture archiving and communication systems, tailored for either clinical or research purposes [26], is important. A user-friendly interface with clear instructions for various actions is needed for that. Alternatively, the dataset can be distributed and securely accessed through platforms like the cancer imaging archive [90, 91].

## Discussion

In recent years, numerous vendors have entered the medical imaging market with AI products to assist clinicians, and even though external validation might have been performed in a limited form in some cases [92], generalizability issues persist with CE-marked or FDA-cleared models, depending on the end-users clinical context. While recommendations on reducing biases exist [3, 59, 74, 77, 93], they do not provide a foolproof guarantee against it. Besides this, AI companies most often do not disclose what data were used exactly to train their models making it hard to compare the training data to the data used in the local clinical setting.

To deal with the absence of benchmark datasets, this publication provides valuable insights for creating such datasets, selecting relevant performance assessment metrics, and considerations on how AI software can be integrated into the clinical workflow. By addressing these aspects, the implementation of AI in radiology has the potential to become more reliable, effective, and ethically sound, ultimately leading to improved patient outcomes. Moreover, recent initiatives like the European Cancer Imaging Initiative (EUCAIM), a federated European digital infrastructure, will result in a large-scale, high-quality dataset ideal for benchmarking [43].

From a stakeholder perspective, choosing an AI software is a non-trivial task since it requires considering parameters like its diagnostic or prognostic performance, interpretability, usability, error rate, integrated workflow, turnaround time, etc., as well as providing services concerning maintenance, post-market surveillance, etc. [94]. Even though there exist publicly available imaging datasets, these cannot be used for validation of AI software since vendors might have used part of that dataset to develop their algorithm and therefore, if this dataset is used for validation it will result in overestimation of the true performance of the algorithm. Caution should also be taken during inference to apply the same preprocessing steps as those used during the training of the developed algorithm. Moreover, caution should be given to the fact that the equipment and the acquisition methods constantly improve (e.g., photon counting CT [95]) and benchmark datasets might end up being outdated at the point of release or some time afterward. Other limitations include the need for an expert opinion to establish the reference standard and possibly the fine-tuning of the parameters of the algorithm that might be required to fit those of the benchmark dataset. At last, the recent AI act in the EU [73] poses new challenges in the adaptation and use of AI solutions in clinical practice that should be considered.

Prior to creating a benchmark, it is important to consider the task in which this dataset would be used. Efforts should be made towards creating more benchmark datasets since they are essential for the validation of AI software before it can be used in clinical practice. Furthermore, a direct comparison of the performance of different vendors on those datasets would allow clinicians to decide which software performs better on a given task.

## Conclusion

In this paper, we provided detailed recommendations regarding benchmark dataset creation, aiming to assist researchers, clinicians, and data scientists in creating high-quality benchmark datasets that are reliable, diverse, and representative of real-world medical data. Ultimately, we believe that the creation of benchmark datasets will facilitate the development of more effective AI models by increasing trust in them, and potentially lead to improved patient outcomes and better healthcare delivery.

## Data Availability

No dataset or any other information can be shared.

## Abbreviations

AUC: Area under the curve
AUC-ROC: Area under the receiving operating characteristic curve
AI: Artificial intelligence
DL: Deep learning
EU: European Union
FL: Federated learning
GDPR: General data protection regulation
IoU: Intersection over Union
NPV: Negative predictive value
